# Fabricating Graphene-Based Molecular Electronics via Surface Modification by Physisorption and Chemisorption

**DOI:** 10.3390/molecules30040926

**Published:** 2025-02-17

**Authors:** Zhi Li, Keying Guo, Chengjie Yin, Yanan Li, Stijn F. L. Mertens

**Affiliations:** 1 Anhui Province Key Laboratory of Specialty Polymers, Anhui Province Engineering Technology Research Center of Coal Resources Comprehensive Utilization, School of Chemical Engineering and Blasting, Anhui University of Science and Technology, Huainan 232001, China; guokeying2023@163.com (K.G.); cjyin@aust.edu.cn (C.Y.); liyanan@mail.ustc.edu.cn (Y.L.); 2 Department of Chemistry, Energy Lancaster and Materials Science Lancaster, Lancaster University, Bailrigg, Lancaster LA1 4YB, UK

**Keywords:** graphene, surface modification, physisorption, chemisorption

## Abstract

Graphene, a one-atom-thick sp^2^-hybridized carbon sheet, has enormous potential for fabricating flexible transparent electronics due to its unique electronic and mechanical properties. However, the intrinsic lack of a band gap, the low reactivity, and the poor solubility of pristine graphene have largely hindered wide-ranging applications so far. One of the most attractive ways to resolve these issues is to modify the graphene surface through molecular physisorption or chemisorption. In this review, we summarize the recent progress in fabricating graphene-based molecular electronics through manipulating small functional molecules on the graphene surface towards chemical reactivity adjustment, molecular doping, and band gap opening via non-covalent and covalent interactions, and draw attention to challenges and opportunities. We also suggest future research directions for graphene-based molecular electronics.

## 1. Introduction

Graphene, a single sheet of sp^2^-hybridized carbon atoms bonded in a honeycomb lattice, was first successfully identified as a thermodynamically stable monolayer film by Novoselov and Geim in 2004 [1]. Since then, this thinnest known material has attracted tremendous scientific interest from both fundamental and applied points of view, due to its remarkable optical [2], electronic [3,4], mechanical [5,6], and thermal properties [7], which outperform most existing materials. Most significantly, the energy dispersion is linear near the six Dirac points, at which the graphene’s conduction and valence bands intersect [8]; thus, graphene can act as a zero-band-gap semiconductor or semi-metal. Obeying a linear dispersion relation, electrons can travel like massless relativistic particles [9], which allows ultra-high data processing speed in graphene-based transistors, and yields aptitude for high-frequency applications in demonstrating record-high cutoff frequencies, maximum oscillation frequencies, and voltage gain [10]. As a result, considering the continuous demand of size downscaling and performance improvement of modern electronic devices, graphene is expected to replace or at least complement metal or silicon-based electrodes in nanoscale electronic devices [11,12,13,14,15]. However, the pristine graphene material has some drawbacks, such as a zero band gap, low reactivity, weak electrochemical activity, easy agglomeration, and poor solubility and processibility, all of which limit the application of graphene. For example, the poor solubility in most solvents caused by the high cohesive interactions makes graphene hard to process, which greatly hinders its practical use [16,17]. Secondly, graphene’s properties strongly depend on the number of graphene layers [18,19,20]. The strong π–π interactions and van der Waals forces between graphene layers often result in the formation of irreversible agglomerates or can cause restacking to form multilayer graphene or graphite [21]. Thirdly, the intrinsic lack of a band gap in pristine graphene restricts its use in logic operations, as it prevents the current flow from being turned off, leading to substantial off-state current leakage and a non-saturating driving current [14]. Another technical problem of integrating graphene into transistors lies in the difficulty to deposit a gate dielectric insulator layer on graphene. Within the graphene lattice, each carbon atom is covalently bonded to three adjacent atoms through σ-bonds, leaving the remaining electrons strongly coupled in the *p*_z_-orbitals and thus forming a giant delocalized π-bonding system, which makes the graphene surface electrically conductive but chemically inert. Combining its inertness and hydrophobicity, pristine graphene is not an ideal substrate for the growth of polar insulators such as SiO_2_, HfO_2_, and Al_2_O_3_ [22,23,24,25]. The advantages and challenges of single-layer pristine graphene are summarized in Table 1.

**Scheme 1:**
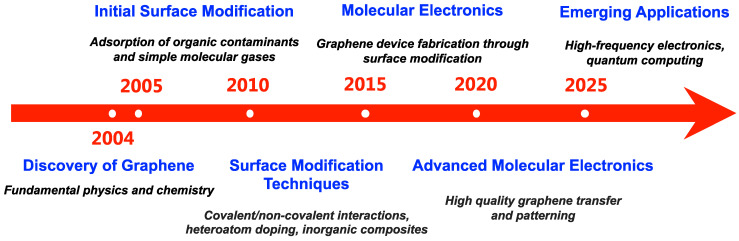
Roadmap of graphene-based molecular electronics via surface modification.

To solve the above-mentioned problems, the molecule-based surface functionalization of graphene holds opportunities to tailor its intrinsic properties and enrich its functionalities for the use in an array of different applications, such as supercapacitors [26,27], dye-sensitized solar cells [28,29], field-effect transistors (FETs) [30,31,32], biosensors [33,34], and so on. The surface functionalization of graphene can be achieved via covalent and non-covalent methods, either by the chemisorption of reactive molecules that form covalent bonds or by the physisorption of organic molecules or SAMs. Modern graphene-based molecular electronics are commonly fabricated based on a bottom-up approach, through which organic molecules are randomly adsorbed or arranged into SAMs via physisorption or chemisorption on graphene as electronic components or wires in electric circuits. Organic molecules have many advantages for the fabrication of molecular electronic devices such as their high degree of uniformity and atomic precision, the rich diversity of molecular species, and versatility of functional groups. Molecular electronics offers a promising way for the further miniaturization of IC devices in the sub-10 nanometer regime. Scheme 1 shows the evolution of graphene-based molecular electronics via surface modification.

In this review, we focus on the fundamental aspects of the chemical functionalization of graphene via chemisorption or physisorption methods and discuss the state of the art in graphene-based molecular electronics that can be fabricated using these methods or a combination thereof, without replicating the perspective of multiple excellent earlier reviews, which, however, were mostly limited to non-covalent modification [35,36]. Finally, we discuss the challenges and perspectives to bring graphene-based molecular electronics to fruition, and highlight promising emerging applications.

## 2. Graphene-Based Molecular Electronics: Physisorption

Due to its “surface-only” nature, graphene is an ideal substrate for surface functionalization with physisorbed molecular species for tailoring its electronic and chemical properties such as building reactive molecular architectures [37,38,39,40], molecular doping [41,42,43], and band gap opening [44,45] towards applications in graphene-based molecular electronics. The large surface area and the presence of delocalized π electrons make graphene strongly susceptible to adsorption [46]. Benefiting from the weak non-covalent interactions (e.g., van der Waals (vdW) force [47], π–π stacking [48], hydrogen bonding [49], and electrostatic forces [50], many of which can be modulated electrochemically [51,52]) between molecules and a graphene substrate, surface functionalization via physisorption is attractive as it allows for precise degree and spatial control over functionalities, and can form uniform, pinhole-free, and flexible SAMs of organic molecules by passing across the defective sites in the graphene surface, such as step edges [25,53,54]. Most importantly, such a functionalization method commonly causes no damage to graphene’s crystal structure and the extended π-conjugation system, and thus no degradation of its unique intrinsic properties [55,56].

### 2.1. Physisorption: Surface Reactivity

Field-effect transistors (FETs) are the functional heart of modern semiconductor devices. To keep decreasing their size, as reflected in Moore’s law, many studies have aimed at integrating graphene into FETs [57,58,59,60]. A critical step in fabricating a high-performance FET is the atomic layer deposition (ALD) of an ultrathin, uniform, and pinhole-free dielectric layer. However, the quality of the dielectric layer by direct ALD on pristine graphene is compromised due to its chemical inertness and hydrophobicity [61,62]. Therefore, considerable efforts have been directed towards activating the graphene surface and improving the resulting ALD layer quality.

**Figure 1:**
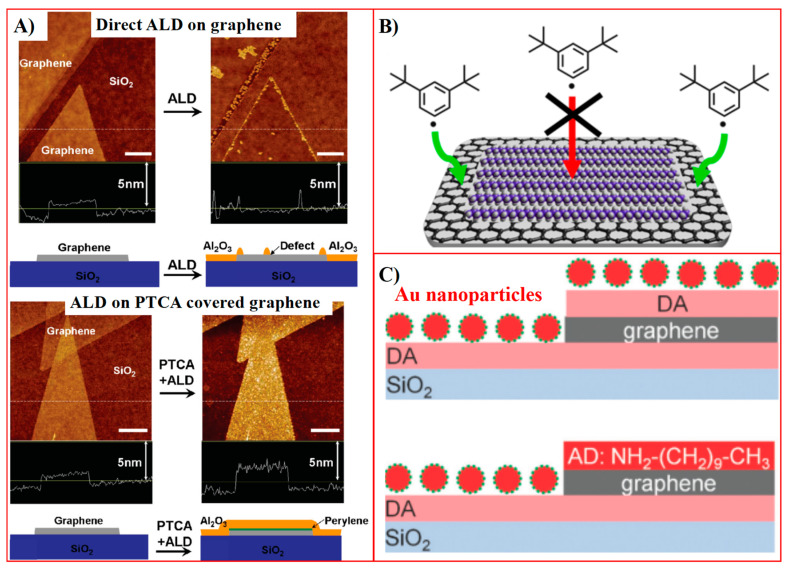
Tuning of graphene surface chemical reactivity via physisorption. (**A**) Comparison of AFM images of direct ALD of Al_2_O_3_ on pristine graphene and on PTCA-covered graphene, showing enhanced surface reactivity of graphene. Reproduced with permission from ref. [57]. Copyright 2008, American Chemical Society. (**B**) Reduction in graphene surface reactivity using SAM of alkane molecules. Reproduced with permission from ref. [59]. Copyright 2017, Royal Society of Chemistry. (**C**) Prevention of gold nanoparticle attachment on graphene using 1-aminodecane (DA) monolayer. Reproduced with permission from ref. [60]. Copyright 2012 Wiley-VCH.

In a very early attempt, graphene was functionalized with 3,4,9,10-perylene tetracarboxylic acid (PTCA) to improve its surface chemical reactivity [57]. The PTCA molecules can easily self-assemble on graphene through π–π stacking into intact, ordered functional monolayers for further ALD of uniform, pinhole-free high-κ dielectric layers. The experimental results show that no ALD of Al_2_O_3_ occurs on the basal plane of the unfunctionalized graphene due to the absence of dangling bonds or surface functional groups, but that Al_2_O_3_ preferentially appears on the defective areas such as the edges, pentagon–hexagon pairs, or vacancies. By comparison, a dense, uniform coating of the PTCA functional layer allows the successful deposition of homogeneous Al_2_O_3_ dielectric film of 2.8 ± 0.2 nm in thickness and 0.33 nm in mean roughness over a 2.5 µm × 2.5 µm area. This approach paves the way for integrating ultrathin high-κ dielectrics into modern electronics with the help of a non-destructive functionalization process by the physisorption of organic molecules. Later on, Alaboson et al. [58] used a 10,12-pentacosadiynoic acid (PCDA) monolayer to improve the chemical reactivity of epitaxial graphene (EG) for templating ALD growth of oxide patterns with sub-10 nm lateral resolution (Figure 1A). Under UHV conditions, PCDA arranged into single-layer one-dimensional arrays that fully functionalize graphene, which subsequently results in templated ALD of ZnO and Al_2_O_3_ because of the preferential reaction between the ALD precursors and the functional groups of the PCDA monolayer. Preserving the molecular-scale 1D PCDA pattern requires the adjustment of precursor–molecular functional group interactions and the overall stability of the physisorbed PCDA monolayer. Both the careful selection of the ALD chemistries and ultraviolet-induced cross-linking of the PCDA monolayer can improve stability of the PCDA template. Conversely to increasing the surface reactivity of graphene, SAMs can also act as a protective layer to decrease its surface reactivity. Li et al. [59] demonstrated that monolayers of densely packed n-pentacontane significantly reduced the surface reactivity of graphene, avoiding the covalent attachment of aryl radicals to its basal plane (Figure 1B). Moreover, Long et al. [60] proved that a 1-aminodecane (Figure 1C) passivating layer prevented a graphene surface from binding gold nanoparticles due to the weak interaction between the alkane tail and the gold nanoparticles.

### 2.2. Physisorption: Molecular Doping

Especially crucial for developing graphene-based molecular electronics is the control of the type and density of charge carriers in graphene [63]. Depending on the direction of the interfacial electron transfer between molecules and graphene, molecules can accordingly act as n-type or p-type molecules. Generally, the adsorptions of gasses [64,65], alkali metal atoms [66,67,68,69], and organic molecules [70,71,72], as well as the substrate underneath [73,74,75,76], are known to be able to dope graphene. Among these, molecular doping via the physisorption of organic molecules offers vital advantages such as precise spatial control over molecular ordering, doping type, and density, whereas the other methods often cause nonuniform doping, may introduce defects or the degradation of graphene properties, or may result in doping that is not reproducible between batches. In particular, aromatic molecules, such as tetrafluorotetracyanoquinodimethane (F4-TCNQ), perylene-3,4,9,10-tetracarboxylic-3,4,9,10-diimide (PTCDI), and its precursor perylene-3,4,9,10-tetracarboxylic-3,4,9,10-dianhydride (PTCDA) have been widely used in organic semiconductors, as the π electrons in these molecules can sufficiently interact with the delocalized π electrons in graphene through π–π stacking during self-assembly, proving to be an effective way to guide the alignment of a stable molecular network and thus precisely control graphene’s electronics properties.

**Figure 2:**
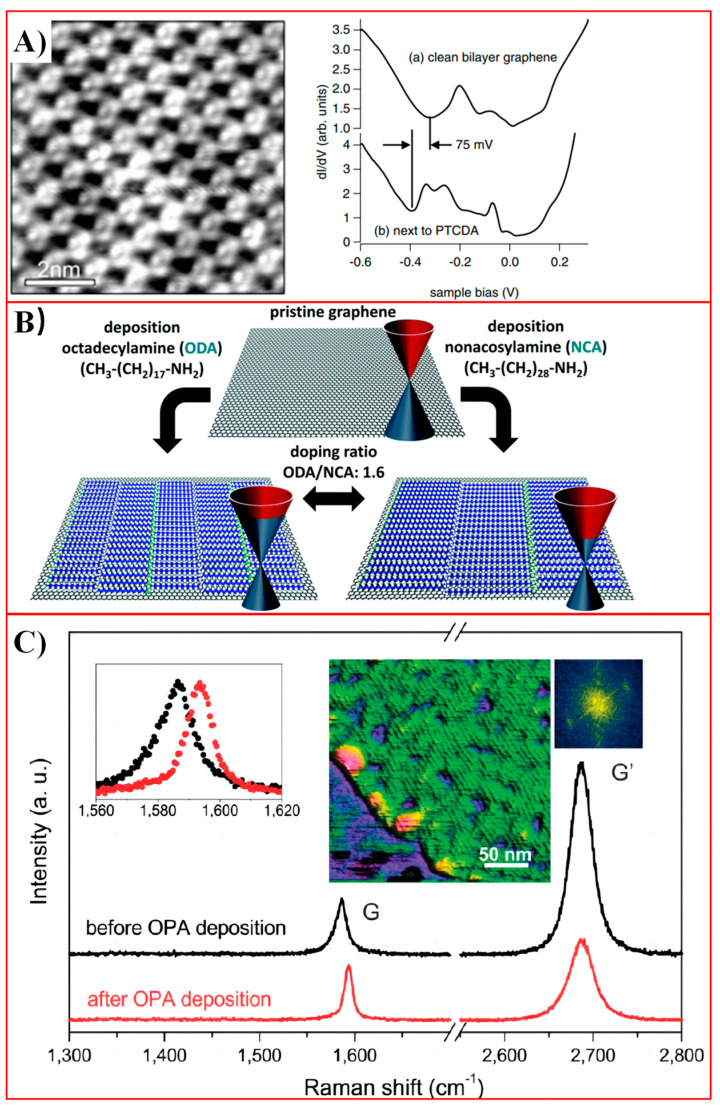
Molecular doping of graphene via physisorption. (**A**) An STM image of SAM of PTCDA on a bilayer graphene surface and comparison of scanning tunneling spectra of bare bilayer graphene and bilayer graphene neighboring the PTCDA monolayer. Reproduced with permission from ref. [37]. Copyright 2008, John Wiley & Sons. (**B**) Tunable doping of graphene by controlling the density of dopant moieties on the surface using self-assembled networks. Reproduced with permission from ref. [78]. Copyright 2016, Royal Society of Chemistry. (**C**) Raman spectra obtained from the same region of a monolayer graphene flake before (black curve) and after (red curve) the deposition of OPA molecules. Reproduced with permission from ref. [79]. Copyright 2011, Royal Society of Chemistry.

Many studies have investigated the functionality of the archetypal π-conjugated molecule PTCDA (the condensed form of PTCA) by physisorption on EG on SiC (0001). The very first study focused on probing the molecular-scale structure and the corresponding electronic properties of PTCDA monolayers on bilayer graphene on a SiC substrate with scanning tunneling microscopy (STM) and spectroscopy (STS) at low temperature (Figure 2A) [37]. They found that PTCDA molecules can self-assemble into a nonplanar brick-wall structure at a high density continuously across the step edges of the underlying bilayer graphene, different from the behavior observed on HOPG [53], which is of great significance for building high-quality pinhole-free graphene-based molecular electronics. Furthermore, a weak electron transfer process was observed from the PTCDA monolayer to graphene substrate, resulting in an n-type doping with a shift in the Fermi level of 75 ± 20 mV at 4.7 K. This doping effect was confirmed by Huang et al. [53] with high-resolution photoemission spectroscopy at 77 K, while no doping was observed at room temperature.

Apart from aromatic molecules, long alkanes bearing functional groups can also tune the doping degrees of graphene. Self-assembled oleylamine (OA) on graphene leads to an n-type doping of graphene based on the electron transfer from the amino group [77]. A uniform monolayer of OA molecules with lamellar structure was visualized by STM and AFM under ambient conditions. The graphene electronic structure was dependent on the surface density of n-type doping OA molecules, and was confirmed by electric transport measurements of back-gated graphene FETs, showing a negative shift in the charge neutrality point by −16 V for the first and −28 V for the second deposition cycle. Additionally, adjusting the periodicity of the functional groups in the SAM of alkylated amines allowed us to tune the doping level of graphene. Phillipson et al. [78] self-assembled octadecylamine (ODA) and nonacosylamine (NCA) on graphene and quantified the n-type doping effect (Figure 2B). As the NCA molecule is roughly 1.6 times longer than that of ODA, the density of the functional groups in the ODA monolayer is expected to be 1.6 times higher than in the NCA monolayer. Experimental values confirmed that the charge carrier concentration in the ODA layer was 1.59 times higher compared to NCA, in excellent agreement with the alkyl chain length ratio of NCA and ODA. The doping effect of ODA-functionalized devices was also 1.52 times higher than that of NCA-functionalized ones based on transport measurements in graphene FETs. By a similar method, Prado et al. [79] managed to form p-type-doped graphene using SAMs of octadecylphosphonic acid (OPA) and tetradecyl phosphonic acid (TPA) with the phosphonic acid groups as electron acceptors (Figure 2C). The degree of p-type doping was characterized by the blueshift in the G-band in the Raman spectrum of the functionalized graphene.

### 2.3. Physisorption: Band Gap Opening

**Figure 3:**
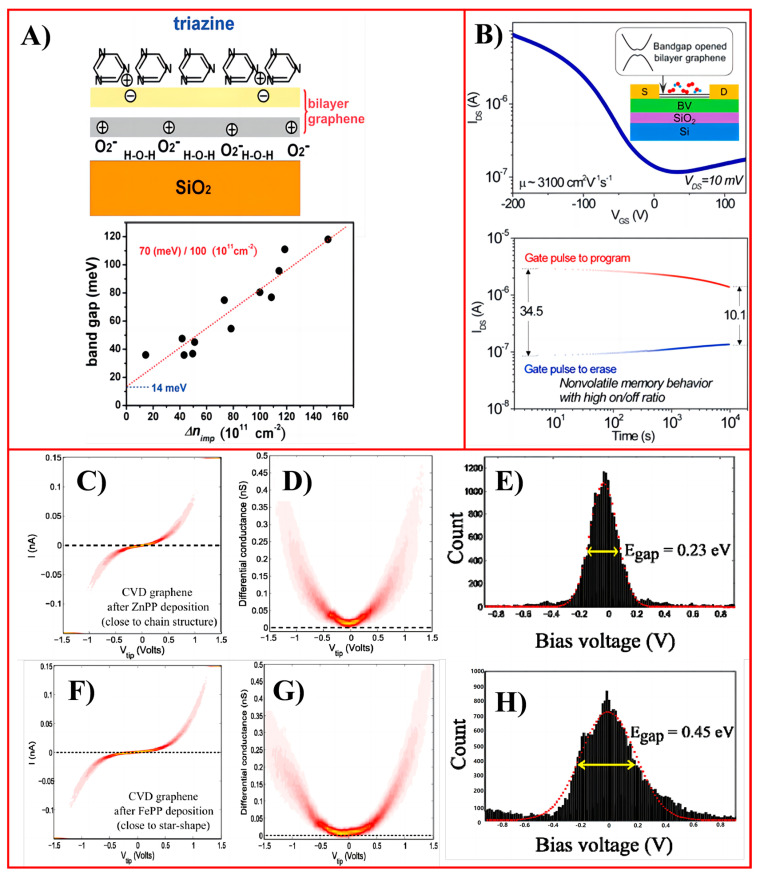
Band gap opening of graphene via physisorption. (**A**) On/off current ratios for bilayer graphene devices before and after treatment with triazine modification and schematic charge distribution after triazine modification on graphene. Reproduced with permission from ref. [85]. Copyright 2011, American Chemical Society. (**B**) Nonvolatile memory cell with a bilayer graphene channel and source/drain electrodes on BV/SiO_2_ (300 nm)/Si, and corresponding I/V curve and retention test (+/−150 V gate pulse for red/blue line). Reproduced with permission from ref. [86]. Copyright 2015, American Chemical Society. I–V curves of (**C**) ZnPP- and (**F**) FePP-modified CVD graphene; (**D**,**G**) differential conductance and (**E**,**H**) Gaussian fit for band gap estimation at low bias. Reproduced with permission from ref. [88]. Copyright 2013, Scientific Research Publishing Inc.

Before bringing graphene into FET nanodevices as a key material, the induction of an electronic band gap remains a pressing issue to solve. Band gap engineering has been studied in single-layer and bilayer graphene using the physisorption of organic molecules. Similarly to the single-layer case, bilayer graphene also behaves like a gapless semiconductor, while applying an electric field across the two graphene layers can induce a band gap. Several theoretical studies have predicted the feasibility of band gap opening of single-layer and bilayer graphene via the physisorption of organic molecules [45,80,81,82,83,84]. Experimentally, Zhang et al. [85] demonstrated a simple but effective method to fabricate an ambient-stable, molecule-decorated bilayer graphene transistor with a moderate on/off current ratio (Figure 3A). The idea is to promote a charge distribution asymmetry within the graphene bilayers by decorating the top layer of graphene with triazine (n-type) and the bottom layer with atmospheric oxygen/moisture (p-type). The resulting band gap scales linearly with the degree of doping with a slope of 70 meV/10^13^ cm^−2^. Upon the molecular decoration, it observes a 3–6-time bigger on/off ratio of bilayer graphene, with a 111 meV gap at maximum dopant concentration. Similarly, Lee et al. [86] fabricated a chemically conjugated bilayer graphene FET with environmentally stable benzyl viologen and oxygen/moisture as the dopants of the bottom and top layers (Figure 3B), producing a 352 meV gap and on/off ratio of 76.1 without significantly degrading the on-current over time. However, in neither of these studies did the band gap reach the desired minimum of 400 meV [87]. Identifying effective molecular dopants to maximize the on/off ratio of graphene FETs and enlarge the band gap remains an active area of research, which for single-layer graphene so far has been seldom successful, possibly due to suboptimal molecular stacking [85]. Nevertheless, Arramel et al. [88] succeeded in opening a band gap larger than 400 meV in monolayer graphene by choosing metal-containing porphyrin molecules as the dopants. They found that physisorbed zinc protoporphyrin (ZnPP) molecules were able to induce a 230 meV band gap in graphene grown on Ni, based on the statistical analysis of STS measurements (Figure 3C–E). A pronounced improvement of the band gap to 450 meV was achieved by replacing the zinc protoporphyrin with iron protoporphyrin, suggesting that the metallic character of the porphyrin determines the graphene–molecule interaction and therefore the band gap opening (Figure 3F–H).

## 3. Graphene-Based Molecular Electronics: Chemisorption

Different from a fragile physisorbed functional layer that is susceptible to operating or environmental conditions (e.g., humidity and temperature), chemisorption (i.e., forming covalent bonds) provides a robust way for graphene functionalization, with long-period stability under various chemical environments. Furthermore, compared to the physisorption method, chemisorption on graphene can more significantly change graphene properties, such as wide band gap opening, and introduce diverse functional groups. Owing to its unique structure, the delocalized π-bonding system renders the graphene surface quite inert. Therefore, highly reactive reagents, such as diazonium-generated radicals, must be used to allow the formation of covalent bonds. Covalent graphene functionalization also transforms locally the sp^2^-hybridized carbon atoms within the graphene lattice into sp^3^-hybridized carbons. Initially, graphene functionalization via chemisorption focused on solution-exfoliated graphite to improve its processability such as dispersion in solvents and anti-aggregation [16,89,90,91]. The covalent functionalization of the basal plane and/or edges of graphene has been reported to improve its thermal [92,93,94], mechanical [95,96], electrical [97,98,99], and optical properties [100,101,102,103], which, as a result, strongly enhances the potential applications of graphene. Recently, the covalent functionalization of graphene on standard substrates such as SiC, SiO_2_, etc., has moved into focus for exploring potential applications of graphene in electronic devices, since theoretical calculations predict that covalent bonding with other atoms or functional groups can open a wide band gap (0.64–3 eV) [104]. Graphene covalent modification has been conducted with various methods, including radical reactions [105,106,107], cycloaddition reactions [108,109], and the introduction of foreign atoms (e.g., hydrogenation [110,111,112] and halogenation [113,114,115]) with different reactive chemical sources such as aryl diazonium salt, hydrogen, or halogen plasma. Among these highly reactive reagents, aryl diazonium molecules have been particularly popular due to their flexibility of substituents, availability, and ability to endow graphene with stable and versatile functionalities. The first attempt of graphene covalent modification using diazonium chemistry was conducted by Haddon’s group [107]. In this research, the authors reacted 4-nitrophenyl diazonium tetrafluoroborate with EG grown on SiC wafers and proposed that a rapid electron transfer from graphene to the diazonium salt occurred, converting the latter into an aryl radical with the release of N_2_. The aryl radical then reacts directly with the sp^2^-hybridized carbon atoms in graphene, forming a covalent bond. Aryl radicals can be generated by the chemical or electrochemical reduction and thermal or light treatment of the corresponding aryl diazonium salts. The presence and extent of graphene covalent functionalization can be determined by Raman spectroscopy [106]. The appearance of a D-band in the Raman spectrum is sensitive to the presence of sp^3^ defects in the graphene lattice, in this case, the formation of covalent bonds between the functional moieties and the graphene substrate. The intensity of the D-band with respect to the G-band scales with the number of such defects associated with covalent functionalization. Therefore, the intensity ratio I_D_/I_G_ can be used to estimate the degree of functionalization. Importantly, removal of covalently bound groups from graphene can be achieved with nm-resolution through tip-induced nanolithography or thermal annealing, and restores all properties of pristine graphene [116,117], making chemisorption on graphene more reversible than previously thought.

### 3.1. Chemisorption: Surface Reactivity

**Figure 4:**
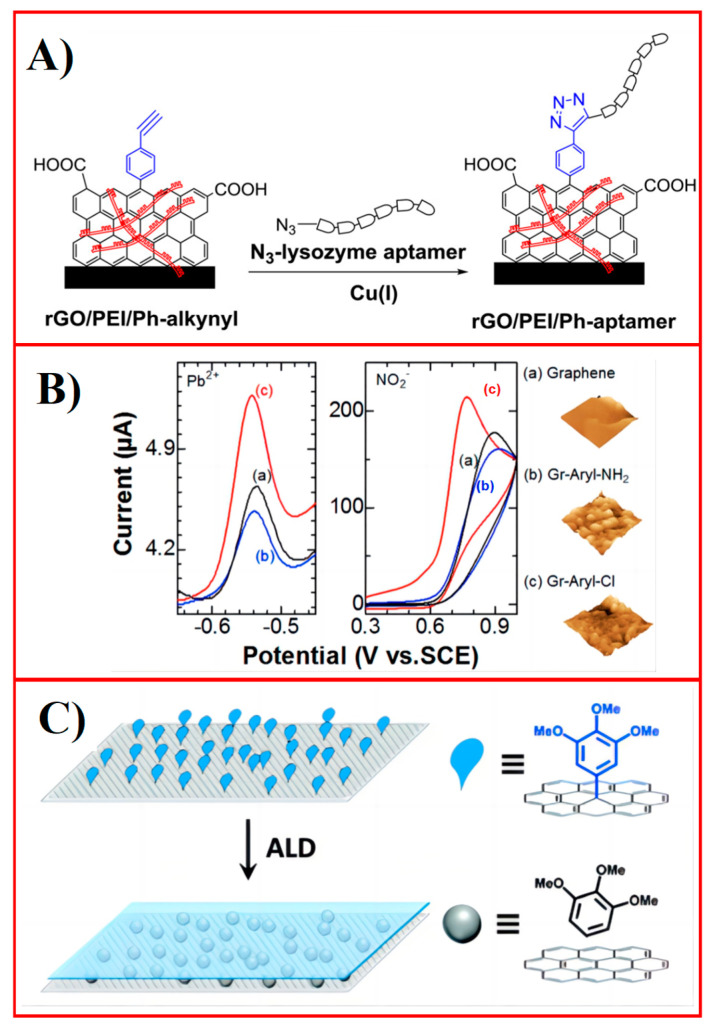
Adjustment of graphene surface chemical reactivity via chemisorption. (**A**) Scheme of lysozyme sensor by click chemistry on covalently modified graphene. Reproduced with permission from ref. [119]. Copyright 2017, American Chemical Society. (**B**) Electrochemical grafting of GNP with various aryldiazonium salts for selective and sensitive monitoring of charged species. Reproduced with permission from ref. [121]. Copyright 2016, American Chemical Society. (**C**) Templating of alumina ALD by covalently modified graphene, followed by restoration of full sp^2^ graphene surface by controlled bond breaking. Reproduced with permission from ref. [124]. Copyright 2021, Royal Society of Chemistry.

The chemical and physical properties (e.g., hydrophilicity, hydrophobicity [118]) of a surface directly depend on the composition and structure of this surface, and on the presence and density of any functional groups. Graphene offers multiple valuable properties for fabricating electrochemical biosensors, including fast electron transfer, excellent electrical conductivity, acceptable electrochemical stability, high specific surface area, and good biocompatibility. Particularly for biosensing applications, the chemical functionalization of graphene with specific functions is critical for facilitating the precise binding of the analyte and avoiding nonspecific interactions and interference. The covalent modification of graphene with aryl diazonium has been demonstrated as a powerful tool in biosensor design, by improving its surface reactivity and accommodating a stable receptor layer onto the transducing graphene substrate. Wang et al. [119] demonstrated a method of covalently functionalizing reduced graphene oxide with various phenyl diazonium precursors, which can further function as ultrasensitive sensing platforms. For example, Figure 4A shows how azide-terminated lysozyme aptamers were clicked onto the –C≡CH functional groups anchored on graphene for detecting lysozyme levels in patients who suffer from inflammatory bowel disease with a 200-femtomolar detection limit and a linear range up to 20 picomolar without signal amplification. Eissa et al. [120] presented the assembly and characterization of an immunosensor by immobilizing an ovalbumin antibody on a -COOH-modified graphene electrode. The resulting biosensor is ultrasensitive for detecting ovalbumin with a linear concentration range from 1.0 pg·mL^–1^ to 100 ng·mL^–1^, and 0.9 pg·mL^–1^ detection limit. Qiu et al. [121] functionalized graphene nanoplatelets (GNPs) with diverse aryldiazonium molecules using a potentiodynamic technique (Figure 4B). Upon adjusting the chemical nature and number of terminal groups (–Cl, –NO_2_, or –NH_2_) in the covalently attached aryl layer, the interfacial properties of the electrodes can be modified accordingly. The resulting functionalized graphene nanoplatelets allowed selective and sensitive monitoring of both cations and anions such as Pb^2+^, NO_2_^−^, and SO_3_^2−^ (Figure 4B). In a recent paper, we demonstrated a one-step process to prepare a β-cyclodextrin-modified graphite electrode, and demonstrated the selective detection of analytes that form inclusion complexes with this supramolecular host against a hundredfold higher background of molecules that do not [122]. Beyond sensor development, this approach is invaluable for preparing materials, which requires precise spatial positioning of functional moieties and efficient current collection, for instance, in photoelectrodes or electrocatalysis.

Covalently anchored functional groups can also be used for modulating the growth of electrocatalysts and thus modifying their morphologies, physical properties, and corresponding electrochemical behaviors. Li et al. [123] explored the feasibility of controlling the shape and the corresponding electrocatalytic behavior of in situ-grown metal–organic frameworks (MOFs) using the covalently functionalized graphene as the template. They found that the MOF was arranged into an octahedral shape on graphene with Ni^2+^/ Co^2+^ (1:1) as the metal nodes and 2,2-bipyridine-5,5-dicarboxylic acid as the organic linker, while it was transformed into nanoflowers with ‘desert rose’ morphology when graphene was covalently anchored with high-density carboxylic groups, which work as the nucleation sites with high surface reactivity promoting the in situ growth of MOFs. Therefore, the resulting MOF owns a large number of active sites, and improved electric conductance, and thus boosted OER activity with a 241 mV overpotential at 10 mA cm^−2^ in the alkaline solution. This approach can effectively control the surface structure and properties of graphene-supported electrocatalysts. Furthermore, similar to the physisorbed organic molecules, a chemisorbed interfacial layer on graphene can be used as a seed layer for the ALD process. Brown et al. [124] grafted tri-methoxy groups on graphene for ALD of Al_2_O_3_ (Figure 4C). A smooth and uniform dielectric layer was observed on the graphene surface based on the scanning electron microscopy (SEM), atomic force microscopy (AFM), and electrical measurements. Importantly, Raman measurements showed that annealing at 150 °C promotes the detachment of aryl groups from the graphene surface during the ALD process, recovering the original sp^2^-hybridized state without damaging the dielectric layer, and thus providing additional nanoscale control and creating further advanced nanoengineering options.

### 3.2. Chemisorption: Molecular Doping

**Figure 5:**
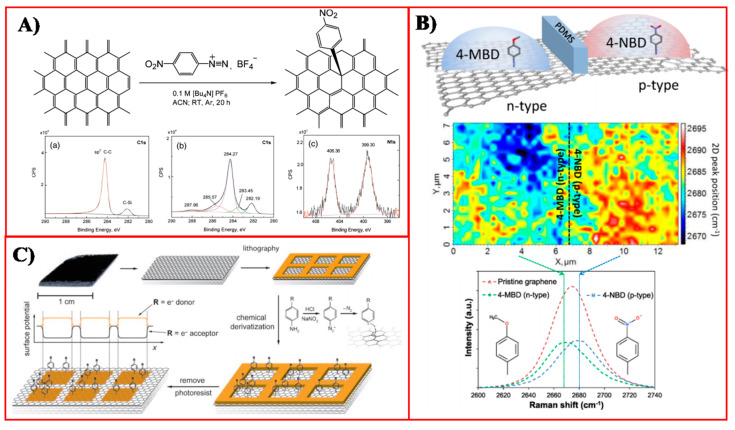
Molecular doping of graphene via chemisorption. (**A**) The scheme of the spontaneous grafting of aryl groups to graphene through the reduction of 4-NBD and XPS spectra of graphene/SiC. Before functionalization: (**a**) C_1s_. After functionalization: (**b**) C_1s_ and (**c**) N_1s_. Reproduced with permission from ref. [107]. Copyright 2009, American Chemical Society. (**B**) A schematic showing the fabrication of the p-n junction on graphene by chemisorption with 4-MBD and 4-NBD on each side of a PDMS membrane. Corresponding Raman mapping and spectra showing the n-type doping and p-type doping effect that arose from the covalent attachment of 4-MBD and 4-NBD on the graphene surface. Reproduced with permission from ref. [126]. Copyright 2013, American Chemical Society. (**C**) The patterned functionalization of an HOPG surface creates periodic surface potential difference: graphene was masked by photo-lithography, followed by exposing the diazonium reagent to unmasked regions. Reproduced with permission from ref. [131]. Copyright 2009, John Wiley & Sons, Inc.

Similar to the physisorption of organic molecules on graphene, the chemisorption of molecules that bear electron-donating or -withdrawing functional groups can also yield n- or p-type doping of graphene to tune its electronic properties. Initially, Bekyarova et al. [107] covalently modified graphene grown on a SiC substrate with 4-nitrobezene diazonium tetrafluoroborate (4-NBD) and claimed a p-type doping effect from the presence of XPS peaks at 285.57 eV (C-N) and 283.45 eV (Figure 5A). Lim et al. [125] later argued that non-covalent functionalization with 4-bromobenzene diazonium (4-BBD) tetrafluoroborate, through partial charge transfer, was responsible for the p-type doping of graphene, rather than covalent functionalization. Subsequently, Bissett et al. [126] systematically investigated the nature of the functional groups and the degree of covalent functionalization on the resulting doping effect. The three different aryl diazonium molecules used were 4-NBD, 4-BBD, and 4-methoxybenzene diazonium (4-MOBD) with either electron-donating or -withdrawing functional groups. The covalent nature of the functionalization was confirmed by the pronounced increase in the defect-caused Raman D-peak, and the doping effect by shifted G and 2D peak positions, caused by the shifted Fermi level at the K point [127,128]. The results showed that covalent attachment of 4-NBD on graphene contributed to upshifted G and 2D peaks. With an increased grafting density by extending the reaction time or improving the surface reactivity, there was a significant increase of the D peak and upshifted G and 2D peak positions due to an enhanced p-type doping effect. Similarly, covalent modification with the bromo-containing molecule also provided a dramatic upshift in the G and 2D peaks, due to the p-type doping effect. In contrast, the covalent modification of graphene with the electron-donating methoxy-containing molecule presented a significant increase in the D peak intensity and with a slight downshift in the G and 2D bands due to the minor n-type doping effect that arose from the nonadiabatic Kohn anomaly [128,129,130]. Based on their findings, the authors managed to fabricate an in-plane p-n junction on a graphene substrate via covalent functionalization with 4-NBD, and 4-MOBD, in order to create two specific areas with p-type and n-type doping effects on strained graphene that was divided using a PDMS mask (Figure 5B). The Raman mapping measurements (Figure 5B) clearly indicated the different doping behaviors between the two specific areas near the interface, which were supported by the shift directions of 2D peak position in Raman spectra (Figure 5B). A similar doping effect was confirmed by Koehler et al. [131] using Kelvin probe force microscopy (KPFM) (Figure 5C). The authors first patterned the graphene surface with a photoresist mask, followed by covalently modifying the unmasked regions of graphene with aryl diazonium molecules with different functional groups. After removing the photoresist mask, the patterned graphene with covalent functionalities was observed by SEM. The KPFM measurements further revealed the difference between the surface potential on modified and pristine graphene surfaces and confirmed that the doping effect was strongly affected by the nature of functional groups. In addition, angle-resolved ultraviolet photoelectron spectroscopy (ARPES) can also be applied as an effective way to quantify the doping level of covalently functionalized graphene based on probing the shift in the Dirac cone of graphene. Ambrosio et al. [132] demonstrated the influence of the diazonium chemistry on the electronic structures on graphene/SiC, through X-ray, Raman, and ARPES measurements. It was proven that the 3,4,5-trimethoxybenzenediazonium (TMeOD) was successfully anchored on the graphene surface on the SiC substrate through covalent bonding. The ARPES measurements showed a downshift in the Dirac cone of TMeOP-grafted graphene due to the slight n-type doping and annealing that can help restore the intrinsic properties of graphene due to the desorption of dopants.

### 3.3. Chemisorption: Band Gap Opening

**Figure 6:**
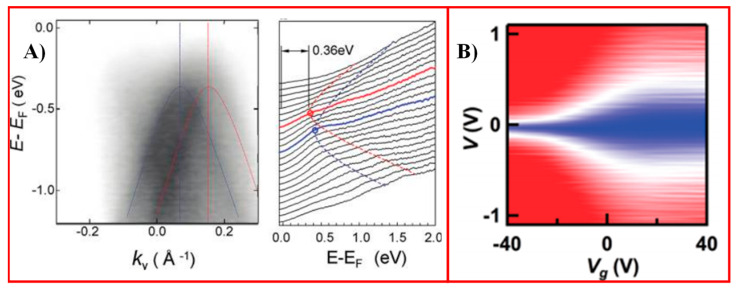
Band gap opening of graphene via chemisorption. (**A**) The band gap in nitrophenyl-functionalized EG measured using ARPES showing a band gap opening of 0.36 eV; d_I_/d_V_ vs. bias V and V_g_ measured on 4-NBD-functionalized EG at T = 4 K showing an estimation of the band gap around 0.1 eV (Red and blue curves represent the two diffuse bands associated with gapped Dirac cones). Reproduced with permission from ref. [138]. Copyright 2010, American Chemical Society. (**B**) d_I_/d_V_ vs. bias V and V_g_ at T = 4 K. Reproduced with permission from ref. [106]. Copyright 2011, American Chemical Society.

Band gap opening by chemisorption is also feasible, since it can generate sp^3^ centers. Therefore, the delocalized electrons can be effectively modified and thus alter the band properties of graphene. Compared to the mild physisorption method, graphene functionalization by chemisorption creates a significant distortion in the geometry and π-clouds of graphene because of the alteration of the hybridization of carbon atoms from sp^2^ to sp^3^, and thereby, the energy band structure of graphene. Two different mechanisms are proposed for band gap opening of covalently functionalized graphene: (1) create a large band gap (1–2 eV) near the sp^3^ carbon centers; (2) generate a tiny gap (100 meV) in the sp^2^ lattice between adjacent sp^3^ clusters [133]. Covalent modification via the hydrogenation of graphene [112,134,135] produced a wide-gap semiconductor, while fluorination [136,137] produced an insulator. Nevertheless, such modification processes are aggressive, thermally unstable, and highly energetic processes that are incompatible with standard semiconductor technology [106]. The covalent functionalization of graphene with diazonium chemistry is a more promising way for band gap opening due to its simple implementation and compatibility with wafer-scale heterogeneous integration and the clearly demonstrated doping effect it produces. Haddon’s group achieved band gap opening by the covalent functionalization of exfoliation and EG on SiC with 4-NBD characterized by Raman and ARPES [138]. A pronounced D-band was observed for both graphene types in the Raman spectra after covalent attachment of 4-NBD, due to the introduction of sp^3^ carbon. In addition, a band gap of ∼0.36 eV was probed by the ARPES measurements upon covalent modification (Figure 6A). However, the electron mobility and device conductance decreased based on the transport measurements. Following this, they found a ∼0.1 eV gap at 4 K under the same covalent modification (Figure 6B) [106]. Although a theoretical 2 eV gap can be achieved based on DFT simulations [104], some limitations exist in practical cases. For example, physisorbed aryl oligomers formed as a by-product of diazonium reduction can block the graphene surface from further covalent functionalization [139]. This effect leads to a low-density modification of graphene, which is far away from the 25% concentration of covalent modification sites for opening a bulk band gap in the electronic structure of graphene [140]. Therefore, optimizing the covalent modification procedure is essential to improve the grafting density and open a sufficient bulk band gap in graphene. Furthermore, avoiding inhomogeneous attachment of aryl groups (on suspended graphene) and charged impurities (on G/SiO_2_) can also dramatically promote graphene band gap opening [141].

## 4. Graphene-Based Molecular Electronics: Physisorption vs. Chemisorption

The physisorption of organic molecules often yields self-assembled molecular architectures with long-range ordering and a high degree of functionality without degrading graphene’s intrinsic properties, yet suffers from limited stability due to its high sensitivity to the changes in operational conditions or the ambient environment. In contrast, chemisorption with reactive reagents can significantly influence the electronic properties of graphene and provide more stable functionalities, at the expense of a significant degradation of its charge carrier mobility, induction of electron–hole mobility asymmetry, and reduction in the minimum conductivity [142]. The advantages and disadvantages of physisorption and chemisorption methods toward graphene functionalization are summarized in Table 2.

Calculations based on density functional theory (DFT), the parameterized tight-binding model, and real-space Kubo–Greenwood formalism predict that structures with nanostripes can be created on covalent nanopatterning of graphene, leaving electron transport unaffected and confined in the resulting quasi-one-dimensional semiconducting channels, while allowing tunable opening of a graphene band gap with high electron mobility within the channels. In addition, the spatially controlled patterning of covalently attached functional moieties onto graphene opens a new domain for tailoring properties in confined graphene regions, such as spatially confined adjustment of hydrophilicity–hydrophobicity, catalytically or optically active domains, anchoring group reservoirs for molecular recognition, and patterned electron conductivity [143]. Therefore, spatially controlled covalent patterning may be the solution for the effective functionalization of graphene without degrading its intrinsic properties by combining the advantages from both the physisorption and chemisorption methods.

**Figure 7:**
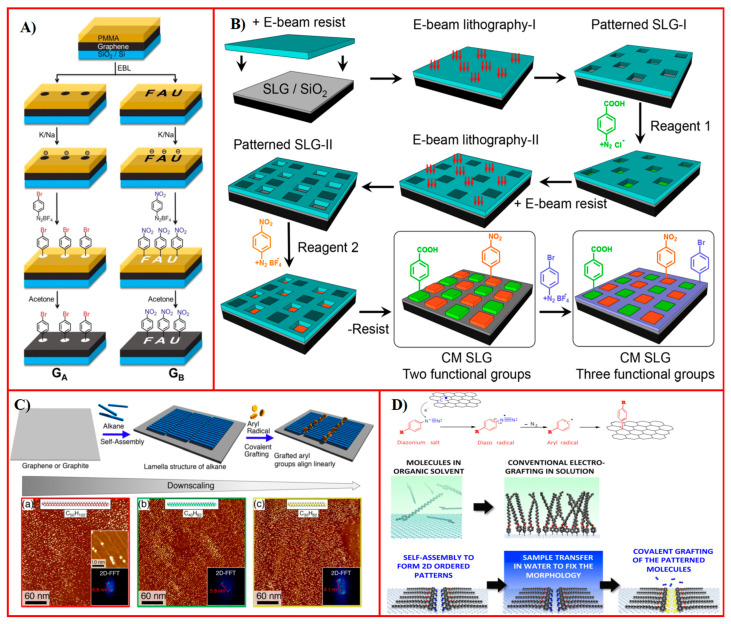
Covalent pattering of graphene using a combined technique. (**A**) Sequential steps for covalently patterned graphene. Reproduced with permission from ref. [144]. Copyright 2020, WILEY-VCH Verlag GmbH & Co. KGaA, Weinheim. (**B**) Multi-component diazonium patterned graphene using the electron beam lithography technique and self-limiting diazonium chemistry. Reproduced with permission from ref. [145]. Copyright 2021, American Chemical Society. (**C**) SAMs of alkanes with adjustable periodicities (**a**) C_50_H_102_, (**b**) C_40_H_82_ and (**c**) C_30_H_62_ as templates to form covalent arrays and corresponding STM images with Fourier transforms. Reproduced with permission from ref. [146]. Copyright 2018, American Chemical Society. (**D**) The electrochemical reduction of pre-assembled p-(n-octadecyloxy)benzenediazonium for covalent patterning of graphitic surfaces. Panel adapted with permission from ref. [148]. Copyright 2016, American Chemical Society.

In earlier research, Koehler et al. [131] demonstrated covalent patterning of graphene by using a removable photoresist mask as the physisorbed template for directing aryl diazonium attachment via electrochemical reduction. According to the chemical nature of the introduced aryl diazonium molecules, the covalently patterned graphene shows spatially confined electronical properties in the micrometer range with a surface potential polarization and gate-induced changes in conductivity. Following a similar strategy, Wei et al. [144] conducted an efficient laser-writing protocol to covalently pattern graphene on SiO_2_/Si at the micrometer scale (Figure 7A). The authors initially covered the graphene surface by a thin film of physisorbed polymethylmethacrylate (PMMA) with a desired pattern. Afterwards, the uncovered graphene regions were negatively charged with a Na/K alloy ion beam, followed by being exposed to positively charged aryldiazonium cations for a covalent reaction. Micrometer-resolution patterns filled with covalently attached aryl diazonium molecules were successfully constructed, after removing the excess reagent and physisorbed template. The recovery of graphene can be achieved by thermal annealing at 400 °C to its original state and thus a complete write/store/erase cycle can be realized to manage the chemical information on graphene electronics. González et al. [145] accomplished covalent pattering of graphene with multiple functional regions using three diazonium salts (Figure 7B). Electron beam lithography was used to form a first pattern on PMMA pre-covered graphene. Subsequently, the first diazonium salt was grafted within the first pattern by chemical activation with ascorbic acid, followed by removing the unreacted reagents and the first template. Afterwards, the second component was patterned on graphene by repeating the procedure in the unmodified areas. Avoiding overlap of each pattern is essential to this experiment and can be achieved using alignment markers in the SiO_2_ substrate. Patterning a third diazonium component was completed on the remaining graphene surface. Moving forward, adapting to the development of modern graphene electronics will require the exploitation of molecular techniques for the shrinkage of covalent patterning on graphene, as pattern dimensions approach the sub-micrometer scale or less.

In addition, SAMs themselves can be effective templates to covalently pattern graphene at the nanometer resolution, and locally protect graphene from radical attack [59]. Linear molecular templates were fabricated by Tahara et al. [146] using SAMs of n-alkanes to guide covalent patterns on graphitic surfaces with precise control of lateral periodicity from 4 to 7 nm, depending on their length (Figure 7C). Later, the authors further succeeded in directing covalent pattering of graphene in hexagonal alignment with a precise control on the lateral periodicity from 2 to 3 nm by employing porous molecular templates, which were formed by self-assembly of alkoxy-substituted dehydrobenzo[12]annulene (DBA) derivatives (DBA-OCns) [147]. These findings may enable fabricating graphene-based molecular electronics with technology nodes within sub-3 nm regions. Finally, Xia et al. [148] demonstrated a two-step procedure of graphene covalent patterning with a lateral periodicity of 3.8 nm by self-assembly of programmed diazonium molecules with long alkoxyl chains, yielding ordered linear patterns prior to electrochemical activation for in situ covalent attachment (Figure 7D). This method paves a way to a simple yet versatile and well-controlled route for covalent nanopatterning of graphene.

## 5. Conclusions and Outlook

The use of graphene in molecular electronics is alluring, as the material possesses several unique and exceptionally desirable electrical and other properties. However, its intrinsic lack of a band gap, poor solubility in most solvents, tendency to self-aggregate, and low chemical reactivity significantly hinder its use in practical applications. In this review, we have focused on the chemical functionalization of graphene via the physisorption or chemisorption of molecular units, and how this enables chemical reactivity adjustment, doping level tuning, and band gap opening. While chemisorption has often been regarded as detrimental to some of the desired properties of graphene, as it effectively introduces defects in its structure, the modification can be selectively reversed by tip-induced nanolithography or thermal annealing, adding powerful options to the growing toolbox of graphene functionalization and, prospectively, unprecedented high-tech applications.

Challenges that remain are, for instance, poor reproducibility of graphene monolayer growth and the resulting devices, which may be caused by the adsorption of impurities, water molecules, and the presence of defects during growth, transfer, or processing. Also, precise control over the doping level of graphene remains elusive. Hence, there is a pressing need to establish effective protocols for producing reproducible high-quality graphene monolayers and achieving in situ functionalization with accurate control over the density and location of molecular modifiers. Machine learning may help to analyze complex data from these devices, optimize their design, and predict their behavior under various conditions [149]. Future developments may rely on hitherto largely unexplored phenomena, including multiphoton effects [149], the extreme sensitivity of graphene’s properties to mechanical strain [150], or electrochemically switchable mobility or crystallinity of molecules on a graphene surface [151]. Turning challenges into successful outcomes will require even deeper understanding of graphene’s chemistry, and creativity in exploring new methods to functionalize its surface. At any rate, combining the merits of molecular physisorption and chemisorption on graphene may go a long way towards these goals.

## Figures and Tables

**Table 1:** Advantages and challenges of monolayer pristine graphene.

ADVANTAGES	CHALLENGES
Atomically thin	Lack of band gap
~97.7% optical transparency	Low chemical reactivity
~200,000 cm^2^/V s electron mobility	Poor solubility
~1 TPa mechanical strength	Easy agglomeration
~5000 W/mK RT thermal conductivity	Poor processability

**Table 2:** Advantages (+) and disadvantages (−) of graphene functionalization via physisorption and chemisorption. Note that both approaches can be chemically reversible, albeit under different experimental conditions.

Physisorption	Chemisorption
(+) Mild reaction conditions	(−) Harsh reaction conditions
(+) Zero graphene degradation	(−) Nonzero graphene degradation
(+) Reversible modification	(+) Reversible modification
(−) Weak interaction	(+) Strong bonding
(−) Limited functionalization effect	(+) Significant functionalization effect
(−) Metastable, fragile	(+) Stable, durable

## Data Availability

Not applicable.
